# HOPE-trial: hemiarthroplasty compared to total hip arthroplasty for displaced femoral neck fractures in the elderly-elderly, a randomized controlled trial

**DOI:** 10.1186/s12891-015-0763-3

**Published:** 2015-10-19

**Authors:** Olof Sköldenberg, Ghazi Chammout, Sebastian Mukka, Olle Muren, Hans Nåsell, Carl-Johan Hedbeck, Mats Salemyr

**Affiliations:** Department of Clinical Sciences, Division of Orthopaedics, Karolinska Institutet at Danderyd Hospital, S-182 88 Danderyd, Sweden; Department of Surgical and Perioperative Science, Umeå University, S-901 85 Umeå, Sweden

**Keywords:** Femoral neck fracture, Hemiarthroplasty, Total hip arthroplasty, Harris hip score, Randomized clinical trial, Octagenerians

## Abstract

**Background:**

A femoral neck fracture (FNF) is a common cause of suffering and premature death in the elderly population. Optimizing the treatment for improved outcome and a reduced need for secondary surgery is important both for the patient and the society. The choice of primary total or hemiarthroplasty in patients over eighty years are controversial. We hypothesized that total hip arthroplasty has an equal or better outcome in patient-reported outcome compared with hemiarthroplasty.

**Methods/Design:**

A prospective, randomized, single-blinded trial will be conducted. We will include 120 patients, 80 years of age and over with an acute (<36 h) displaced femoral neck fracture. The patients will be randomized in a 1:1 ratio to either total hip arthroplasty or hemiarthroplasty. The primary endpoints are Harris hip Score and EQ-5D. Secondary endpoints include pain measured with visual analogue scale, surgical time, reoperations, complications and radiological measurement of erosion in patients operated with hemiarthroplasty. Follow-up will be performed postoperatively after three months, 1, 2, 4 and 10 years.

**Discussion:**

To our knowledge, this is the first randomized controlled trial comparing total hip arthroplasty and hemiarthroplasty for displaced femoral neck fracture in patients age 80 years and over.

**Trial registration:**

Clinicaltrial.gov: NCT02246335

## Background

How femoral neck fractures in aged patients should be treated are determined by the degree of displacement, age, functional demands, surgeon preference and risk for complications associated with cognitive function and degree of physical fitness [1–7]. The main methods of choices are internal fixation, hemiarthroplasty and total hip arthroplasty. Internal fixation is the main alternative for young patients with displaced intracapsular fractures and in frailest elderly patients who are not medically fit for prosthesis surgery [2, 8]. Most surgeons seem to recommend that hemiarthroplasty is the preferred treatment for elderly patients with low functional demands in the absence of arthritic changes in the hip [9]. However, despite extensive research during the last decades and a number of randomized controlled studies that has been published comparing the types of arthroplasty in the treatment of displaced femoral neck fractures - the question remains if there is any advantage in replacing the healthy acetabulum with a prosthetic cup in the healthy, cognitive intact elderly patient [10–15]. A number of published studies have suggested that total hip arthroplasty may produce better hip patient reported outcomes compared with hemiarthroplasty. However, the majority of studies conducted are made for a subgroup of patients that are active and living in their own homes without cognitive alteration. The study settings are, with few exceptions [12, 14, 15], made up of a relative large population of patients below 80 years of age. Nevertheless, even a patient with low demands seems to benefit from a total hip arthroplasty. Reported advantages of hemiarthroplasty over total hip arthroplasty are a reduced rate of dislocation, shorter operation times including less blood loss and less technically demanding surgery [14-17]. Surgeons favouring total hip arthroplasty rely on the tendency for improved hip function. The longevity and level of activity of todays elderly increases the risk for protrusion of the femoral head in the acetabulum and the need for revision operation. Despite the more extensive surgery during total hip arthroplasty there has not been any detectible difference in mortality in comparison to hemiarthroplasty [1, 18].

**Fig. 1:**
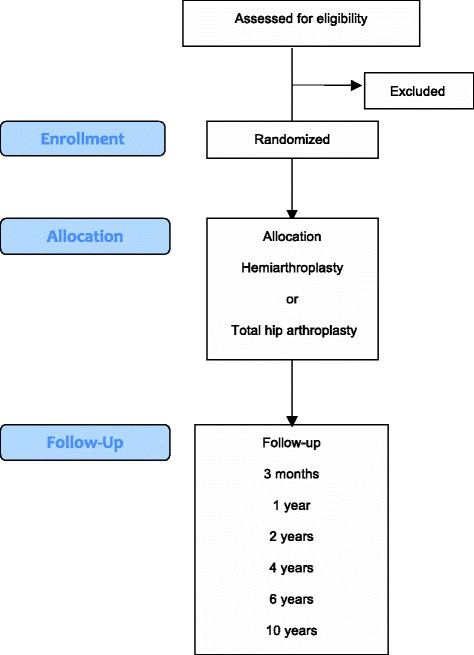
Flow diagram

We hypothesized that a total hip arthroplasty results in superior hip function and health-related quality of life compared to to hemiarthroplasty in healthy and cognitively intact patients aged above 80 years treated for a displaced femoral neck fracture. The secondary outcome measurement includes mortality, complications and reoperations.

## Methods/Design

### Study design

A randomized controlled trial will be conducted. The study will be carried out from 2009–2026 (inclusion period 2009–2016) at the Orthopaedic Department of Danderyd Hospital in collaboration with the Karolinska Institute. The study will be conducted in accordance with the ethical principles of the Declaration of Helsinki. The Ethics Committee of the Karolinska Institute approved the study. The trial is initiated, designed, and performed as an academic investigation and registered at ClinicalTrials.gov (number NCT02246335). The guidelines of the CONSORT Statement will be followed [19].

### Study population

All patients with a femoral neck fracture who are admitted to Danderyd Hospital during the inclusion period will be screened for participation in the study and those who agree to participate and provide written informed consent will be included. The inclusion criteria are an acute displaced femoral neck fracture (Garden stage III or IV) that had been sustained within the previous thirty-six hours, an age of eighty years or more [20], an ability to walk indoors and outdoors with or without the help of walking aids and an absence of cognitive dysfunction (Pfeiffer test 8–10 points) [21]. Exclusion criteria includes patients with osteoarthritis or rheumatoid arthritis in the fractured hip, pathologic fractures, non-walkers, severe cognitive impairment (Pfeiffer test 0–7), and contraindications for surgery by other reasons (ulcers, severe somatic illness, severe bleeding disorder) and those who were unsuitable to participate in the trial for any other reason (i.e. psychiatric disease, alcoholism).

A detailed inclusion and exclusion list is presented in Table 1. The inclusion period is planned from 2009–2016. End of study (EOS) will be at the last included patient’s last visit in 2026 (Fig. 1, Table 2).

**Table 1:** Detailed inclusion and exclusion criteria

Inclusion criteria	
1. Age above 80 years.	
2. Acute (<36 h) displaced femoral neck fracture (Garden ≥3).	
3. Independent walking ability with or without walking aids.	
4. Pfeiffer test 8–10 points.	
Exclusion criteria	
1. Osteoarthritis or rheumatoid arthritis in the fractured hip	
2. Pathologic fractures	
3. Non-walkers	
4. Cognitive impairment (Pfeiffer Test 0–7)	
5. Contraindications for surgery by other reasons (ulcers, severe somatic illness, severe bleeding disorder).	
6. Unsuitable to participate in the trial for any other reason.

**Table 2:** Secondary endpoints

No.	Measurement	Follow-up time
1	Baseline data including height, weight, medical history, physical examination	Screening
2.	Pain Numerical Rating Scale (PNRS)	Screening, 3 months, 1, 2, 4, 6 and 10 years
3.	Complications	Screening, 3 months, 1, 2, 4, 6 and 10 years
4	Reoperations	Screening, 3 months, 1, 2, 4, 6 and 10 years

### Treatments

Half of the patients will receive a cemented hemiarthroplasty with a modular unipolar head. The other half will receive a cemented total hip arthroplasty with a 32-mm femoral head articulating on a cemented highly cross-linked polyethylene acetabular component. The same stem and the same bone cement will be used for both groups. The operations will be performed either by a consultant orthopedic surgeon or by a registrar with assistance from a consultant. A direct lateral approach will be used [22]. The direct lateral approach is performed through a midline incision over the greater trochanter through the subcutaneous fat and the fascia. The anterior one-third of the gluteus medius insertion and gluteus minimus insertion on the tip of greater trochanter are detached. Excision of the anterior hip joint capsule is performed from the base to the acetabulum. The fractured femoral neck is planed and the caput femoris is extracted. During closure of the wound, the detached part of muscle gluteus medius and minimus is re-attached to the trochanter. Prophylactic cloxacillin is administered 30 min before surgery, and the cloxacillin also another 3 times over 24 h postoperatively. Low-molecular weight heparin will be administered for 30 days postoperatively. The patients are mobilized according to a standard physiotherapy program, and full weight bearing with the use of crutches are encouraged. Plain x-rays of the pelvis and hip will be taken postoperatively and at follow-up.

### Randomization and blinding

Patients will be randomized in a 1:1 ratio to hemiarthroplasty or total hip arthroplasty using concealed envelopes in batch sizes of 10. Randomization will be stratified for gender and patients will be blinded for the assigned treatment during the first two years of follow-up. Patients will not be allowed to view x-rays. Ward staff and physiotherapists are aware of allocations but are mindful that patients are blinded and are encouraged not to speak to patients about allocation. The physiotherapy, nor any other in-hospital care, do not differ between the two groups. In addition, the blinding will be controlled for at the 1 and 2 year follow-up where patients will be asked what group they think they belong to. The blinding is ended at the 2-year follow-up and in case of any hip related serious adverse events such as reoperations for any reasons.

### When and how to withdraw subjects from the trial

Should a subject request or decide to withdraw from the study, all efforts will be made to complete and report the observations as thoroughly as possible up to the date of withdrawal. For withdrawn subjects the last post-baseline observation will be carried forward. In a case of withdrawal of full consent, the subject will be followed according to the routine standard follow-up of arthroplasty patients at our institution, including regular clinical examinations and radiographic follow-up visit at the operating surgeon every 2–3 years.

### Endpoints and follow-up

The primary endpoint variable will be patient-reported functional hip status assessed with Harris hip score [23–25] and health related health-related quality of life assessed by the EQ-5D (EuroQoL) at 2 years. EQ-5D uses five dimensions: mobility, self-care, usual activity, pain/discomfort and anxiety/depression [26].

Secondary endpoints include functional scores at the other follow-up times and pain in the operated hip evaluated with VAS (Visual Analogue Scale [27], surgical time and blood loss, erosion of the acetabular cartilage in patients treated with hemiarthroplasty (evaluated according to Baker et al. [11]).

The study period is 10 years. Follow-up will be performed at 3 months, 1, 2, 4, 6 and 10 years. First study results will be reported after the last patient enrolled in the study has been followed for 2 years.

### Data quality assurance

The study progress and study conduct will be monitored before, during and after the study to ensure that ICH-GCP, regulatory requirements, and all aspects of the protocol are followed. A digital case report form (CRF) will be used throughout the study. The medical records and other documents will be reviewed for verification of agreement with data on the CRF. The subject has a right for a protection against invasion of privacy. In this study, each subject will receive a unique identification number, which will be linked to the CRF. The data will then be blinded correspondingly in all data analyses. However, the study monitor, auditor, representative from any regulatory authority, as well as the appropriate Ethical Committee are permitted to review the subject’s primary medical records including laboratory test result reports, ECG reports, admission and discharge summaries, AE and SAE reports occurring during the study.

### Sample size

Prior to the commencement of the study, a two-sided power analysis has been performed. We will test the null hypothesis that the mean Harris hip scores for the two groups will be equal. We assume that a mean difference of 10 points (standard deviation, 15 points) [4] in the Harris hip score is the smallest effect that will be clinically relevant. We calculate that a total of 80 patients (40 in each group) will have a power of 80 % to yield a significant result.

This calculation also allows a 80 % power to prove non-inferiority (non- difference) of the secondary endpoint EQ-5D with a sample of 40 patients in each group with the assumption of an EQ-5D value of 0.73 with standard deviation 0:18. The significance level was set at a conservative 2.5 % (p <0.025) because we have two sample size calculations. We will include 60 patients in each group (120 total) to allow for loss to follow-up.

### Statistics

The analyses will be performed on the basis of the intention-to-treat principle, and all patients who are allocated to either group will be included in the analysis, regardless of actual surgery performed. Descriptive statistics (means and standard deviations) will be used to describe the patient characteristics and outcome variables at the measurement points. We will use the Student’s t-test and Levene’s test for comparison of Harris hips score and EQ-5D between the groups. An analysis of covariance (ANCOVA) of the primary endpoint will also be used to reduce variance, adjusted for exposure variable (total hip arthroplasty/hemiarthroplasty) and stratification (male/female). For subjects that withdrawn from the study before completion, the data from the last observation will be carried forward (imputed). The analyses will be performed with SPSS 22.0 for Windows (SPSS, Chicago, Illinois) statistical software.

## Discussion

### Rationale for the study

Whether to choose hemiarthroplasty or total hip arthroplasty in the treatment of displaced femoral neck fractures in the most elderly and fit patients are often debated. Despite indications that total hip arthroplasty yields a better patient reported outcome, it is plagued with an increased risk for prosthetic dislocation and thus revision surgery in patients with cognitive impairment. The frequency of patients with manifest or impending cognitive impairment increases with age and thus the risk for complications. The previously published studies in the field have a heterogeneity across the included selection of patients and distinct subgroup effects [18]. There is a lack of level one evidence regarding the choice of arthroplasty in the fit most elderly patients.

### Study design

The internal validity of this trial is good due to the strict inclusion criteria, recruitment procedure and rigorous follow-up. However, some difficulties regarding blinding must be mentioned. Postoperative X-rays will reveal the type of allocation for the investigators. However, patients are blinded to their treatment and thus would not affect the patient reported outcome.

The exclusion criteria can affect the external validity and generalizability due to the fact that a large number of patients will be excluded from the trial. However, the exclusion criteria are mainly focusing on excluding patients with either a malignant disease or those with contraindications for either treatment methods.

We have chosen the primary end-point to be evaluated at several follow-up periods postoperatively, to be able to detect differences in rehabilitation and during short to medium term. We expect that the effect of acetabular erosion may influence the results of hemiarthroplasty at follow-up after the 1 year follow-up.

### Patient reported outcome measurements

The trial’s main outcome measurements are Harris hip score and EQ-5D. Both of these are valid measurements and has been used in an number of hip fracture trials.

Although Harris hip score has been questioned due to ceiling effect and may be inferior to WOMAC and HOOS as a disease specific questinnaire, this has not been confirmed in the hip fracture population [28–30]. The use of HHS also makes comparisons to other trials easier and it has been validated for the use in patients with a femoral neck fracture [30].

## Conclusion

The present trial will provide evidence for the future choice of arthroplasty in elderly patients above the age of 80 years with a displaced femoral neck fracture and without cognitive impairment.
